# Resveratrol enhances the inotropic effect but inhibits the proarrhythmic effect of sympathomimetic agents in rat myocardium

**DOI:** 10.7717/peerj.3113

**Published:** 2017-03-30

**Authors:** Jesús Hernandez-Cascales

**Affiliations:** Department of Pharmacology, Faculty of Medicine, University of Murcia, Espinardo-Murcia, Spain

**Keywords:** Resveratrol, Cardiac contractility, Cardiac arrhythmias, Sympathomimetic drugs, Phosphodiesterase inhibtors

## Abstract

**Background:**

Resveratrol is a cardioprotective agent with known antiarrhythmic effects that has recently been shown to inhibit phosphodiesterase (PDE) enzyme activity. Thus, it is possible that resveratrol increases the inotropic effect of sympathomimetic agents, as PDE inhibitors do but, unlike other PDE inhibitors, its effect may not be accompanied by proarrhythmia due to its antiarrhythmic action. This work is aimed to test this hypothesis.

**Methods:**

This is an “*in vitro*” concentration-response relationship study. The effects of noradrenaline, tyramine and isoproterenol, alone or in combination with either resveratrol or with the typical PDE inhibitor 3-isobutylmethylxantine (IBMX), were studied in electrically driven strips of right ventricle or in the spontaneously beating free wall of the right ventricle of rat heart in order to investigate inotropic or proarrhythmic effects respectively. Also, the effects of resveratrol or IBMX on the sinoatrial node rate were examined in the isolated right atria of rat heart.

**Results:**

Resveratrol (10 µM and 100 µM) produces a leftward shift in the concentration-response curves for the contractile effects of noradrenaline, tyramine or isoproterenol and reduces the –log EC_50_ values of these three agents. IBMX produces similar effects. The spontaneous ventricular beating rate was increased by all three compounds, an effect that was further enhanced by the addition of IBMX. In contrast, resveratrol (100 µM) abolished the effects of these sympathomimetic agents on the ventricular rate. Resveratrol (1–100 µM) had no effect on the sinoatrial node rate, while IBMX produce a concentration dependent sinoatrial tachycardia.

**Discussion:**

Taken together, the finding, indicate that resveratrol, like the PDE inhibitor IBMX enhances the contractile effects of sympathomimetic agents but, in contrast to IBMX, it does not enhance their proarrhythmic effect or produce sinoatrial tachycardia. This is most probably consequence of the antiarrhythmic effect of resveratrol which protect against the proarrhythmic effects resulting from PDE inhibition.

## Introduction

Sympathetic nervous activity increases cardiac contractility, mainly through noradrenaline release, which stimulates *β*-adrenoceptors and consequently enhances cyclic adenosine monophosphate (cAMP) production via the stimulatory G protein/adenylyl cyclase pathway (Overgaard & Dzavík, 2008). This is a very important mechanism for maintaining cardiac output and, indeed, drugs which mimic sympathetic nervous activity such as dobutamine, noradrenaline or dopamine are the mainstays for therapy of cardiogenic shock and acute heart failure therapy (Overgaard & Dzavík, 2008). In addition to inotropic effects, cAMP also has arrhythmogenic effects; indeed cAMP-dependent inotropic agents induce cardiac arrhythmias (Lubbe, Podzuweit & Opie, 1992; Lerman et al., 2000). Cyclic nucleotide phosphodiesterases (PDEs) limit the inotropic and proarrhythmic effects of cAMP-producing agents by breaking down cAMP into its chemically inactive product, 5’AMP (Bender & Beavo, 2006). PDE inhibitors, such as amrinone or milrinone, are effective inotropic agents that increase myocardial contractility by inhibiting the breakdown of cAMP within the cell, but induce arrhythmogenic effects (Amsallem et al., 2005). This deleterious proarrhythmic effect counteracts the beneficial inotropic effect of these agents and limits their clinical efficacy. Indeed, the long-term administration of PDE inhibitors to patients with heart failure leads to an increase in mortality that correlates with an increase in ventricular arrhythmias and sudden cardiac death (Packer et al., 1991; Amsallem et al., 2005).

Resveratrol, a polyphenol phytoalexin, present in red wine and grapes, exert cardioprotective effects by pre-conditioning the heart, in addition to having anti-oxidant and anti-inflammatory effects and inhibiting platelet aggregation and having favourable activity on the lipid profile (Liew, 2008; Raj, Zieroth & Netticadan, 2015). Resveratrol has antiarrhytmic effects (Zhang et al., 2006; Chen, Su & Hung, 2007; Chen et al., 2008; Chen et al., 2009; Xin et al., 2010) and has recently been shown to inhibit PDE activity (Park et al., 2012). Thus, it is possible that resveratrol may increase cardiac contractility although, in contrast to other PDE inhibitors, this inotropic effect may not be accompanied by proarrhythmia due to its antiarrhythmic action. Such a hypothesis was thought to merit further investigation, which is the purpose of the present work. Abnormal automaticity is an important mechanism underlying cardiac arrhythmias and cAMP increases the spontaneous firing rate of pacemaker cells (DiFrancesco, 2010). The right ventricle of the rat contains pacemaker cells in the His-Purkinje fibres, which develop spontaneous activity resembling an idioventricular rhythm (Mangoni & Nargeot, 2008), making it a useful experimental model for assessing the proarrhythmic or antiarrhythmic effects of different agents (Hernàndez et al., 1994; Gonzalez-Muñoz et al., 2011). The aims of the present work were to investigate the effects of resveratrol on the inotropic and proarrhythmic effects of the sympathetic neurotransmitter noradrenaline (exogenously applied or endogenously released by tyramine), and on the effect of the *β*-adrenoceptor agonist, isoproterenol. For comparison, the typical PDE inhibitor 3-isobutylmethylxantine (IBMX) was used. Also, the effects of both resveratrol and IBMX were tested in the spontaneously beating right atrium of rat heart to ascertain whether o or not they affect the sinoatrial node, which is the primary pacemaker of the heart. The findings show, for the first time, that resveratrol enhances contractility but inhibits the proarrhythmic effects of sympathomimetic agents without altering the sinoatrial node rate.

## Methods

### Animals

The study was performed in accordance with the European Union Council Directive of 22 September 2010 (2010/63/EU) for the protection of animals used for scientific purposes and reviewed and approved by the Ethical Committee of the University of Murcia (A13150604). Male Sprague-Dawley rats (250–350 g) were used in the experiments. The animals were housed in an animal room illuminated with a 12/12 h light/dark cycle (light from 07:00 to 19:00 h) at 20–22 °C and had free access to food and drinking water. Rats were rendered unconscious instantaneously by cerebral concussion and euthanized by rapid exsanguination, after which the chest was opened and the heart rapidly removed and placed in Tyrode solution of the following composition:136.9 mM NaCl, 5 mM KCl, 1.8 mM CaCl2, 1.5 mM MgCl2, 0.4 mM NaH2PO4, 11.9 mM NaHCO3 and 5 mM dextrose. The right atrium and the free wall of the right ventricle were excised.

Right ventricular strips (10 mm long, 1 mm wide and 0.5 mm thick) were mounted longitudinally between two platinum electrodes in Tyrode solution. The preparations were electrically stimulated for1ms with a GrassSD-9 stimulator (Quincy,MA,USA) at a frequency of 1 Hz and supramaximal (threshold +25%) voltage. Contractions were measured using a GrassFT-03force–displacement transducer (Quincy,MA,USA) (Juan-Fita, Vargas & Hernandez, 2003; Pérez-Schindler et al., 2011). The effects on ventricular automaticity were tested in the spontaneously beating free wall of the right ventricle. For this, its atrial end was fixed to a metallic support and the apical end was attached to a Grass FT 03 force–displacement transducer. No electrical stimulation was applied (Hernàndez et al., 1994; Gonzalez-Muñoz et al., 2011). The right atria was isolated and suspended in an organ bath. The lower end of the right atrium was fixed on a hook and the upper end was connected by a silk thread to an isometric force–displacement transducer (Grass FT-03). Tissues were immersed in a 30 ml organ bath filled with Tyrode’s solution, gassed continuously with 95% O_2_–5% CO_2_ and maintained at 36 °C and pH 7.4 (measured every 15 min with an electrode: Minitrode–Hamilton, Bonaduz, Switzerland). Tissues were allowed to equilibrate for 45–60 min, before drug challenge, under a preload tension of 0.5 g for atria and of 1 g for ventricular preparations. Contractions were recorded and displayed on a computer screen using a Stemtech amplifier (Stemtech Inc., Houston, Texas) and ACODAS software (Dataq Instruments, Inc., Akron, Ohio).

### Experimental protocol

To investigate the effect of exogenously applied or endogenously released catecholamines on ventricular contractility, cumulative concentration-response curves for noradrenaline, tyramine and isoproterenol were determined in the electrically driven strips of right ventricle. Drug concentrations were increased by 0.5 log units, and each concentration was left for 5 min before applying a higher one. concentration-response curves for these agents were also obtained in the presence of 10 µM and 100 µM concentrations of resveratrol which has been shown to exert PDE inhibitory effects (Park et al., 2012). For comparison, interactions of the three sympathomimetic agents with 30 µM IBMX, which effectively inhibits PDE activity in rat ventricular myocardium (Pérez-Schindler et al., 2011; Soler et al., 2015) were studied. Resveratrol or IBMX were left in contact with the tissue for 15 min before construction of the concentration-response curve for each sympathomimetic agent. Only one concentration-response curve for noradrenaline, isoproterenol or tyramine alone or in the presence of either resveratrol or IBMX was determined in the same tissue. Experiments were terminated by increasing the Ca^2+^ concentration to 9 mM, which produced a maximal contractile response, and the results are expressed as percentages of this effect and also in mN.

To study whether resveratrol influences the proarrhythmic effect of sympathomimetic agents, an effective concentration (≈EC_50_ values obtained from inotropic data), of either noradrenaline, tyramine or isoproterenol was applied to the spontaneously beating right ventricle before adding either resveratrol or IBMX five minutes later. Ventricular frequency (beats min^−1^) was calculated as the average rate of the preparation recorded during the incubation period for each agent.

The effects of either resveratrol or IBMX on the sinus node rate were also investigated by determining the concentration-response relationship of each agent in the spontaneously beating right atrium of rat.

### Drugs

Resversatrol, noradrenaline, tyramine, isoproterenol, IBMX, lidocaine and diltiazem were obtained from Tocris Bioscience (Madrid, Spain) and dimethyl sulphoxide (DMSO) from Probus, (Barcelona, Spain).

Resveratrol and IBMX were dissolved in DMSO and Tyrode solution (20% DMSO in Tyrode) and noradrenaline, tyramine, isoproterenol, lidocaine and diltiazem were dissolved in Tyrode solution. This stock solution was diluted in a pre-warmed and pre-aerated bathing solution to achieve the final concentration desired. The drug was added to the organ bath at an appropriate concentration so that the concentration of DMSO in the test solution was less than 0.3%, which did not affect any of the preparations.

### Statistical analysis

The results are expressed as mean values ± SEM. Concentration-response curves were fitted with non-linear regression sigmoidal concentration-response curves and variable slope and –log EC_50_ values were estimated from the concentration-response curves using the GraphPad 5 Software Inc. San Diego, CA, USA).

Student’s *t*-test or one-way analysis of variance followed by the Newman Keuls post-hoc test for multiple comparisons were used. The criterion for significance was that *p* values should be less than 0.05.

## Results

### Inotropic effects

**Figure 1:**
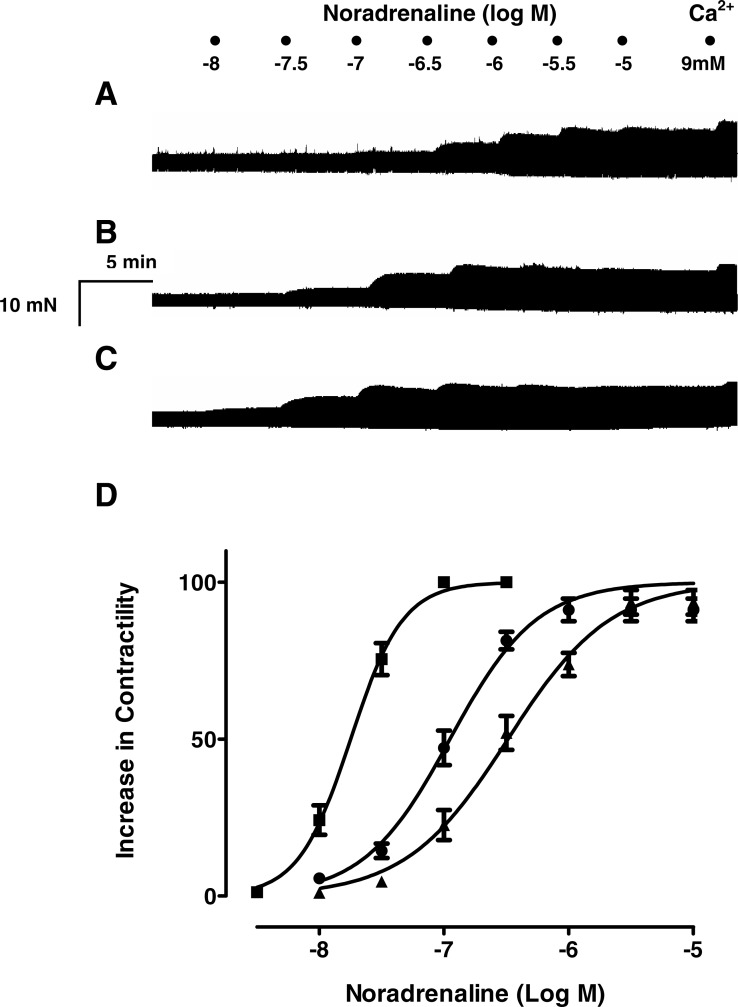
Resveratrol increases the inotropic effect of noradrenaline in rat ventricular myocardium. Representative traces in three strips obtained from the same right ventricle of rat heart, showing (A) the contractile effect of noradrenaline (10-8 to 10-5 M), alone and in the presence of resveratrol 10 µM, (B) and 100 µM (C). (D) Cumulative concentration-response curves for the inotropic effect of noradrenaline alone (▴) and in the presence of resveratrol 10 µM (●) or 100 µM (■), Ventricular strips were electrically driven at 1 Hz and supramaximal (threshold + 25%) voltage. Inotropic responses are expressed as percentage of the effect caused by 9 mM Ca^2+^. Each point represents the mean value ± SEM (vertical bars) of 4–5 experiments.

**Figure 2:**
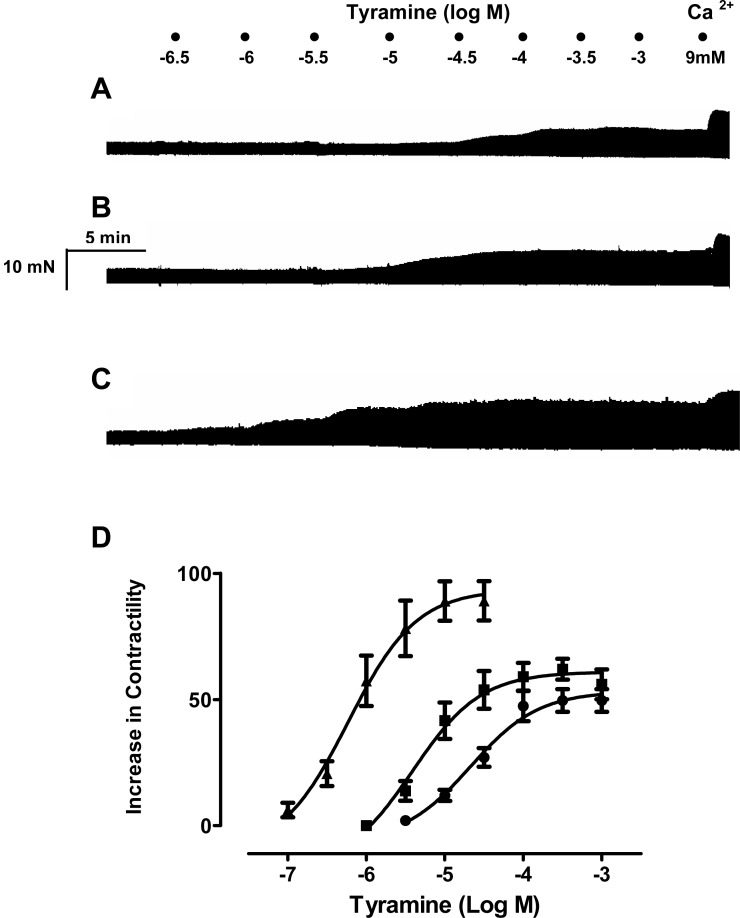
Resveratrol increases the inotropic effect of tyramine in rat ventricular myocardium. Representative traces in three strips obtained from the same right ventricle of rat heart, showing (A) the contractile effect of tyramine (3.10-7 to 10-3 M), alone and in the presence of 10 µM, (B) and 100 µM (C) resveratrol. (D) Cumulative concentration-response curves for the inotropic effect of tyramine alone (●) and in the presence of resveratrol at 10 µM (■) or 100 µM (▴). Further details as in legend to Fig. 1. Each point represents the mean value ± SEM (vertical bars) of 4–6 experiments.

**Figure 3:**
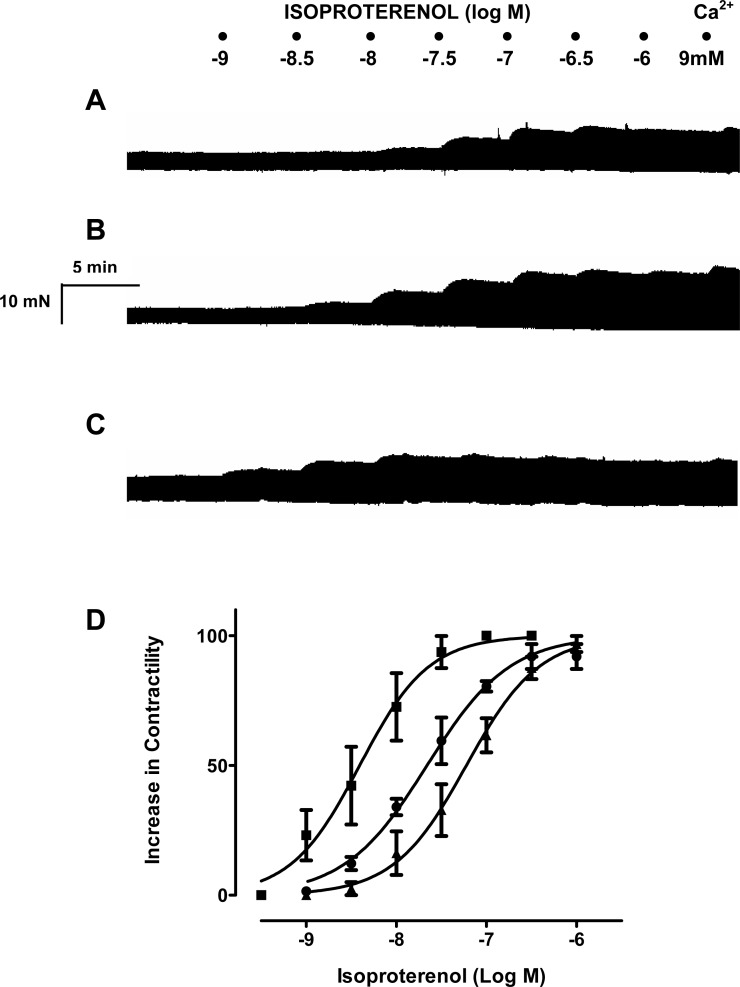
Resveratrol increases the inotropic effect of isoproterenol in rat ventricular myocardium. Representative traces in three strips obtained from the same right ventricle of rat heart, showing (A) the contractile effect of isoproterenol (10-9 to 10-6 M), alone and in the presence of resveratrol at 10 µM, (B) or 100 µM (C). (D) Cumulative concentration-response curves for the inotropic effect of isoproterenol alone (▴) and in the presence of resveratrol at 10 µM (●) or 100 µM (■). Further details as in legend to Fig. 1. Each point represents the mean value ± SEM (vertical bars) of 4 experiments.

**Figure 4:**
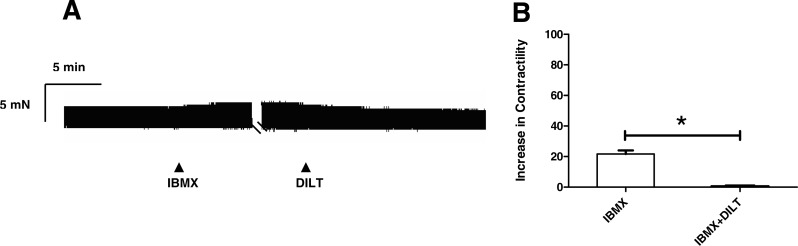
Diltiazem abolish the inotropic effect of IBMX in rat ventricular myocardium. (A) Representative trace showing the effect of IBMX (30 µM) in a rat right ventricular strip. As can be seen, IBMX enhances basal contractility, an effect nullified when 5 µM of the L-type Ca2 + current inhibitor, diltiazem, is added. (B) Effect of IBMX (30 µM) alone and combined with 5 µM diltiazem (IBMX + DILT) in rat right ventricular strips. Inotropic responses are expressed as percentage of basal contractility. Further details as in legend to Fig. 1. Each bar represents the mean value ± SEM of 4 experiments.

**Figure 5:**
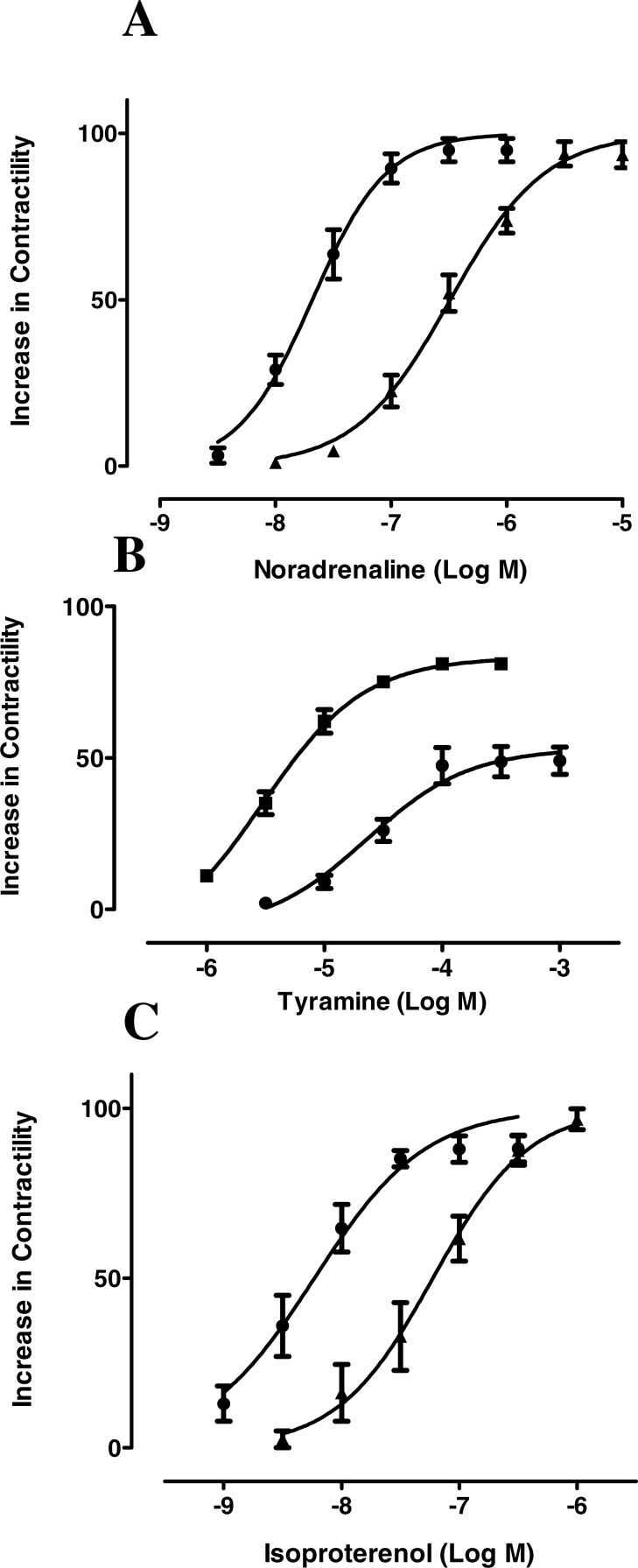
IBMX increases the inotropic effect of noradrenaline, tyramine and isoproterenol in rat ventricular myocardium. Cumulative concentration-response curves for the inotropic effects of (A) noradrenaline, (B) tyramine and (C) isoproterenol alone (▴) or in the presence of 30 µM IBMX (●) in electrically driven rat right ventricular strips. Further details as in legend to Fig. 1. Each point represents the mean value ± SEM (vertical bars) of 4 experiments.

To ascertain whether resveratrol enhances the inotropic effects of endogenously released or exogenously applied catecholamines, concentration-response curves of noradrenaline, tyramine and isoproterenol were constructed in the absence and in the presence of resveratrol. As can be seen in Fig. 1, a 10 µM concentration of resveratrol enhanced the inotropic effect of noradrenaline, and produced a leftward shift in its concentration-response curve, which was further accentuated at 100 µM (Fig. 1). Also, the −log EC_50_ value of noradrenaline was reduced in the presence of 10 µM and 100 µM (Table 1). The inotropic effect of endogenous noradrenaline released by tyramine was also increased by 10 µM and 100 µM resveratrol, which produced a leftward shift of the concentration-response curve for tyramine (Fig. 2) and reduced its −log EC_50_ (Table 1). Similarly, the contractile effect of the sympathomimetic agent isoproterenol was enhanced by resveratrol (10 µM and 100 µM) leading to a leftward displacement in its concentration-response curve (Fig. 3) and a reduction in its –log EC_50_ (Table 1). The 10 µM and 100 µM concentrations of resveratrol were devoid of inotropic effect when applied alone. For comparison, concentration-response curves of noradrenaline, tyramine and isoproterenol were determined in the absence and presence of the PDE inhibitor IBMX (30 µM). In contrast to resveratrol, IBMX on its own increased basal contractility by 21.7 ± 2.3 (*n* = 4). To test whether the inhibitory effect of L-type Ca^2+^ current, which possesses resveratrol (Chen, Su & Hung, 2007) but not IBMX (Li, Himmel & Ravens, 1994), plays a role in this behaviour, the interaction between IBMX and diltiazem, a known inhibitor of L-type Ca^2+^ current, was studied. A 5 µM concentration of diltiazem, which inhibits L-type Ca^2+^ by 50% (Freedman et al., 1984), was seen to abolish the positive inotropic effect of IBMX (Fig. 4, *n* = 4). Like resveratrol, IBMX produced a leftward shift in the concentration-response curves and reduced the −log EC_50_ values obtained for the inotropic effects of these agents (Fig. 5 and Table 1). The vehicle used to dissolve resveratrol or IBMX had no effect on the concentration-response curves for noradrenaline, tyramine or isoproterenol, or their respective −log EC50 values (Table 1).

**Table 1:** −log EC_50_ values for the inotropic effects of Noradrenaline, Tyramine and Isoproterenol in rat ventricular myocardium.

	Noradrenaline	(*n*)	Tyramine	(*n*)	Isoproterenol	(*n*)
Control	−6.5 ± 0.07	5	−4.5 ± 0.11	6	−7.1 + 0.13	4
Vehicle	−6.5 ± 0.17	3	−4.5 ± 0.06	3	−7.2 ± 0.1	3
Resveratrol (10 µM)	−6.9 ± 0.04^*^	5	−5.2 ± 0.13^*^	4	−7.7 ± 0.10^*^	4
Resveratrol (100 µM)	−7.7 ± 0.03^*^^†^	4	−6.1 ± 0.14^*^^†^	5	−8.4 ± 0.23^*^^†^	4
IBMX (30 µM)	−7.7 ± 0.05^*^	4	−5.5 ± 0.08^*^	4	−8.3 ± 0.06^*^	4

**Notes.**

Values are mean ± SEM. *n*, number experiments.

* *p* < 0.05 when compared to control values.

† *p* < 0.05 when compared to Resveratrol (10 µM).

Student’s *t*-test or one-way analysis of variance followed by the Newman Keuls post-hoc test for multiple comparisons.

### Proarrhythmic effects

**Figure 6:**
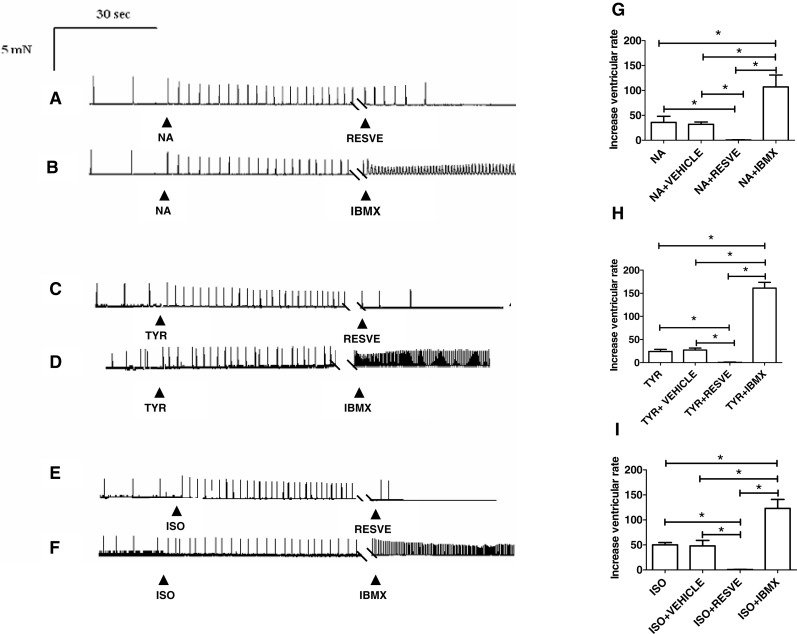
The effects of noradrenaline, tyramine or isoproterenol are enhanced by IBMX but abolished by resveratrol. Representative traces showing the effect of noradrenaline (NA) alone and in combination with (A) resveratrol (RESVE) or (B) IBMX; tyramine (TYR) alone and in combination with (C) RESVE or (D) IBMX and isoproterenol (ISO) alone and in combination with (E) RESVE or (F) IBMX on automaticity in the spontaneously beating isolated right ventricle of rat. Application of a 0.3 µM concentration of NA, 30 µM of TYR or 0.1 µM of ISO (which correspond to their respectives EC50 for inotropic effects), increased the ventricular rate, but 100 µM RESVE abolished spontaneous ventricular contractiliy. In contrast, IBMX (30 µM) further enhanced ventricular frequency. Effects of 100 µM RESVE, 30 µM IBMX or vehicle (VEHICLE) on the increase in spontaneous ventricular rate induced by either (G): noradrenaline (NA), (H): tyramine (TYR) or (I): isoproterenol (ISO). Each bar represents the mean value ± SEM (vertical bars) of 4–5 experiments. (*) *p* < 0.05 between indicated values.

**Figure 7:**
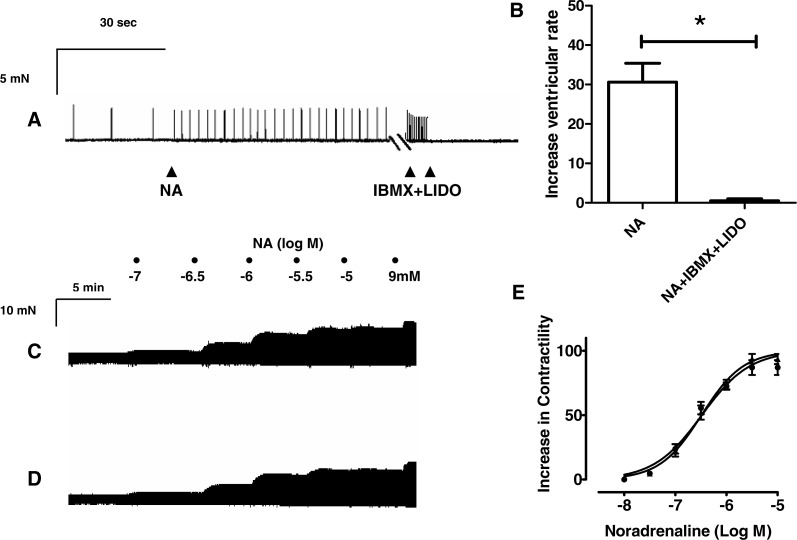
Effects of lidocaine on proarrhythmic and inotropic responses to IBMX and noradrenaline, respectively, in rat ventricular myocardium. (A) Representative trace showing the effect of a concentration 0.3 µM of noradrenaline (NA), which corresponds to its EC50 for inotropic effects, in the absence and in the presence of IBMX alone or combined with lidocaine (LIDO). As can be seen, NA induces an increase in the ventricular rate, which is further enhanced by 30 µM IBMX but abolished when adding LIDO (30 µM) (B) Effects of NA alone or combined with IBMX and lidocaine (NA + IBMX + LIDO) on the spontaneous ventricular rate. Representative traces in two strips obtained from the same right ventricle of rat heart, showing that the contractile effect of noradrenaline (10-7 to 10-5 M), (C) is not modified by the presence of LIDO (30 µM) (D). (E) Cumulative concentration-response curves for the inotropic effect of NA alone (▴) and in the presence of LIDO 30 µM (●). Further details as in legend to Fig. 1. Each point of the concentration-response curves and each bar in (E) represents the mean value ± SEM (vertical bars) of 3–5 experiments.

To investigate whether resveratrol modifies the proarrhythmic effects of catecholamines, experiments were performed in the spontaneously beating isolated right ventricle of rat heart. For this, 0.3 µM of noradrenaline, 30 µM of tyramine and 0.1 µM of isoproterenol, which are their respective EC_50_ values for the inotropic effects, were used. The mean ventricular rate, before addition of any drug was 16.4 ± 3.2 beats min^−1^ (*n* = 35). Figure 6A shows a typical response when noradrenaline was added to a bathing solution containing a spontaneously beating right ventricle. As can be seen, noradrenaline increased the ventricular rate from 7 beats min^−1^ to 38 beats min^−1^, but, after the addition of 100 µM resveratrol spontaneous ventricular activity was abolished. In contrast, IBMX (30 µM) further enhanced the increase in the ventricular rate induced by noradrenaline (from 27 beats min^−1^ to 80 beats min^−1^, Fig. 6B). Several similar experiments were performed and the results are presented in Fig. 6G. To test whether the inhibitory effect of Na^+^ current, which possesses resveratrol (Chen, Su & Hung, 2007) but not IBMX (Egan et al., 1988), plays a role in the abolition of spontaneous ventricular frequency, the interaction between IBMX and the typical inhibitor of Na^+^ current, lidocaine was studied. The addition of a concentration 30 uM of lidocaine, which inhibit Na^+^ current by 50% (Scholz et al., 1998), cancelled the effect of IBMX and abolished the ventricular frequency (Figs. 7A–7B, *n* = 4). However, this inhibitory effect of Na^+^ current did not affect the contractile effect of noradrenaline, which was not modified by lidocaine (Figs. 7C–7E). Like noradrenaline, both tyramine and isoproterenol increased the ventricular rate and these effects were abolished by resveratrol (100 µM) but enhanced by 30 µM IBMX (Figs. 6C–6F and 6H–6I). Interestingly, resveratrol (100 µM) not only abolished the spontaneous ventricular contractions but prevented them from being restored by higher concentrations of noradrenaline (3 µM, *n* = 4), tyramine (100 µM, *n* = 5) or isoproterenol (1 µM, *n* = 5), each of which produced the maximal inotropic response (see Supplemental Information). The resveratrol vehicle (DMSO), failed to alter the spontaneous ventricular frequency (Figs. 6G–6I). A lower concentration of resveratrol (10 µM) was tested but it did not alter the effects of noradrenaline, tyramine or isoproterenol on the ventricular rate.

### Effects of resveratrol and IBMX on the sinoatrial node rate

**Figure 8:**
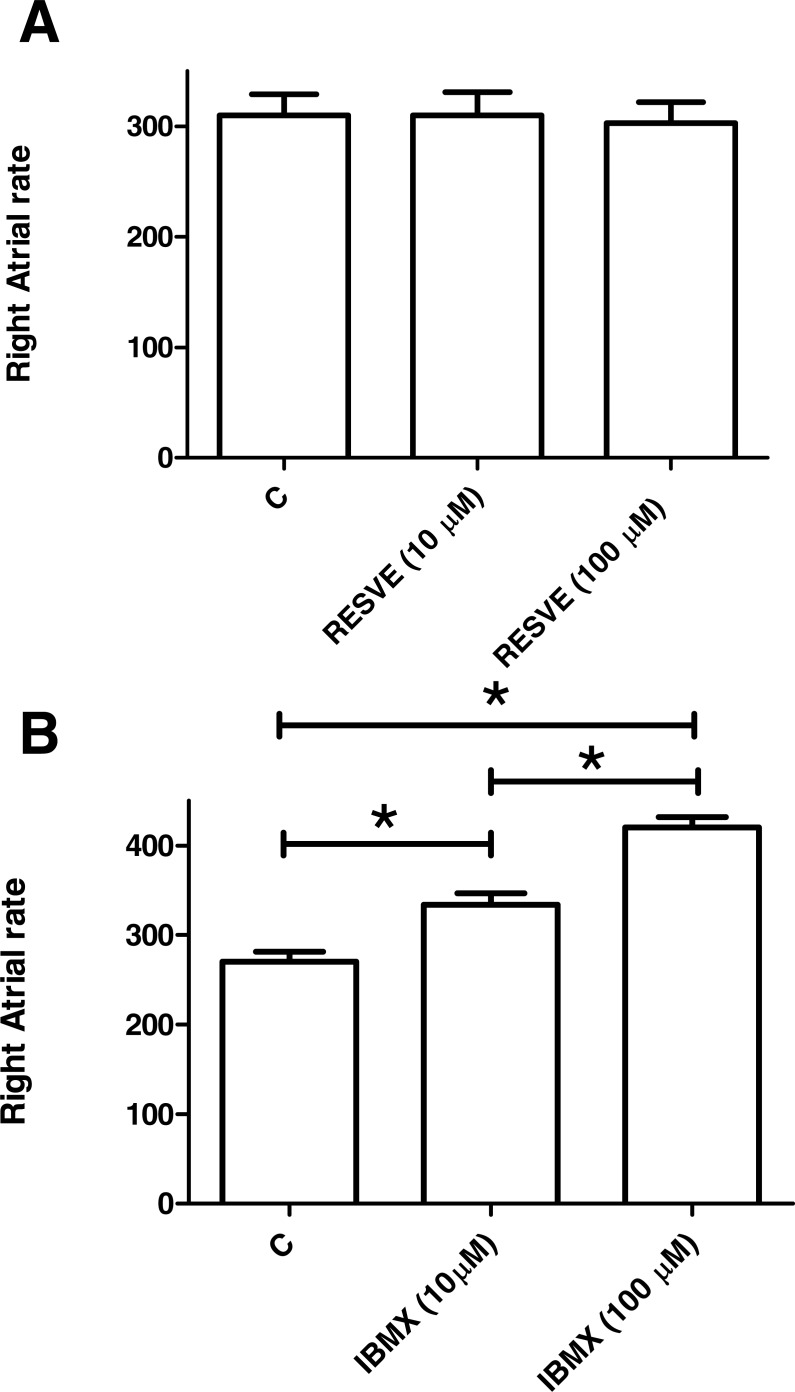
Effects of resveratrol and IBMX on rat sinus node rate. Effects of 10–100 µM resveratrol (RESVE, A) and IBMX (B), on sinus node rate. Each bar represents the mean value ± SEM (vertical bars) of 6–7 experiments. C, control rate. (*) *p* < 0.05 between indicated values.

Resveratrol, at concentrations 1–100 µM, had no effect on the sinoatrial node rate. In contrast, IBMX increased atrial frequency by 22.6 ± 3.7 beats min^−1^ (*n* = 7) at 1 µM, an effect that was enhanced by higher concentrations of the drug (Fig. 8). The vehicle was devoid of any effect on the sinoatrial node rate (see Supplemental Information).

## Discussion

This study shows for the first time that resveratrol increases cardiac contractility but reduces the cardiac automaticity induced by sympathomimetic agents.

The inotropic agents available to date, both, cAMP-dependent such as *β*-adrenoceptor agonists or phosphodiesterase inhibitors, or non cAMP-dependent such as digitalis alkaloids, enhance cardiac contractility but also have proarrhythmic effects (Overgaard & Dzavík, 2008), which limits their clinical effectiveness. Thus, it would be very useful to have available an inotropic drug that produced no proarrhythmic effect or, even better, that had antiarrhythmic effects. Resveratrol is a cardioprotective agent which has been proven to exert antiarrhythmic effects. Indeed, resveratrol reduces ventricular arrhythmias induced by programmed electrophysiological stimulation (Xin et al., 2010) or by proarrhythmic agents such as aconitine or ouabaine in rats and guinea-pigs (Zhang et al., 2006). Also, resveratrol has been seen to protect against ventricular tachyarrhythmias following myocardial infarction induced by coronary artery ligation in rats (Chen et al., 2008) and to suppress the ischemia/reperfusion-induced ventricular arrhythmias in Langendorff-perfused rat hearts (Chen, Su & Hung, 2007; Chen et al., 2009). This is consistent with the results presented here, whereby resveratrol was seen to abolish the proarrhythmic effect of noradrenaline, tyramine and isoproterenol in the experimental model of ventricular automaticity. The antiarrythmic effect of resveratrol is mainly due to its electrophysiological actions (Chen et al., 2008). Indeed, the100 µM concentration used, which abolished ventricular automaticity in the present study, reduces sodium, calcium and potassium currents in rat ventricular myocardium by around 50% (Chen, Su & Hung, 2007) and also inhibits calcium release from the sarcoplasmic reticulum (Liu et al., 2005), which contributes to the enhancement of ventricular automaticity and proarrhythmic effects elicited by catecholamines (Myles et al., 2015).

Recently, it has been demonstrated that resveratrol also inhibits PDE activity (Park et al., 2012). The sympathetic nervous system increases myocardial contractility state through the activation of *β*-adrenoceptors and the consequent enhancement of cAMP production (Overgaard & Dzavík, 2008). PDE enzymes are crucial in regulating cAMP-mediated effects since they provide the only mechanism that degrades cAMP (Bender & Beavo, 2006), and their inhibition enhances the inotropic effects of *β*-adrenoceptor agonists (Juan-Fita, Vargas & Hernandez, 2003). This is consistent with the fact that the PDE inhibitor IBMX enhanced the inotropic effect of noradrenaline, tyramine and isoproterenol in this work. Resveratrol also increased the inotropic effect of these agents. In contrast to IBMX, resveratrol does not enhance basal contractility, probably due to its inhibitory effect on L-type Ca^2+^ current (Chen, Su & Hung, 2007), which contributes to the positive inotropic effect of PDE inhibitors (Li, Himmel & Ravens, 1994). Indeed, the addition of 5 µM diltiazem, which has been seen to inhibit L-type Ca^2+^ current by 50% (Freedman et al., 1984), abolished the inotropic effect of IBMX in our study.

The augmentation of the contractile effect of noradrenaline, tyramine and isoproterenol induced by resveratrol is most probably due to its inhibitory effect on PDE3 and PDE4, which are responsible for most cAMP PDE activity in the rat heart (Mongillo et al., 2004; Rochais et al., 2006), and their inhibition enhances the inotropic effect or noradrenaline in this preparation (Juan-Fita, Vargas & Hernandez, 2003). Indeed, the inhibitory activity of resveratrol on these two isoenzymes is similar to that of IBMX, the IC50 being ∼10 µM for PDE3 and 14 µM for PDE4 in the case of resveratrol (Park et al., 2012) and 3 µM for PDE3 and 14 µM for PDE4 in the case of IBMX (Stoclet et al., 1995). Resveratrol, as well as IBMX inhibit PDE1 with an IC50 of 6 µM and 2 µM for resveratrol and IBMX, respectively (Stoclet et al., 1995; Park et al., 2012). However, in contrast to IBMX, which inhibits PDE2 with an IC50 of 7.6 µM (Stoclet et al., 1995), resveratrol does not inhibit PDE2 (Park et al., 2012). However, although these two isoenzymes, PDE1 and PDE2 are present in the rat heart, they do not limit the contractile effect of noradrenaline in this preparation (Juan-Fita, Vargas & Hernandez, 2003). Our results demonstrate, for the first time, that resveratrol enhance the inotropic effect of the sympathomimetic neurotransmitter noradrenaline, whether exogenously administered or endogenously released by the effect of tyramine, or other sympathomimetic agents such as isoproterenol. This effect of resveratrol is an interesting and novel finding which may have clinical relevance since it is produced at a concentration 10 µM, which is similar to the plasma levels obtained in humans given resveratrol orally (Brown et al., 2010; Van der Made, Plat & Mensink, 2015). In addition to its inotropic effects, cAMP also enhances ectopic automaticity and induces ventricular arrhythmias (Lubbe, Podzuweit & Opie, 1992). This agrees with the results of the present work in which all, noradrenaline, isoproterenol and tyramine enhanced the spontaneous ventricular rate. PDEs restrict this proarrhythmic effect by hydrolyzing cAMP and hence, PDE inhibitors sensitize the heart to the arrhythmogenic effect of catecholamines. This is also consistent with the fact that the PDE inhibitor IBMX further enhanced the increase in the ventricular rate induced by noradrenaline, tyramine or isoproterenol. This proarrhythmic effect resulting from the suppression of PDE activity may have deleterious consequences, particularly for heart failure which is characterized by high sympathetic activity and noradrenaline plasma levels (Viquerat et al., 1985). In fact, the long term administration of PDE inhibitors significantly increase cardiac death, sudden death and cardiac arrhythmias in patients suffering from chronic heart failure (Packer et al., 1991; Amsallem et al., 2005). In contrast to IBMX, resveratrol does not increase ventricular automaticity, but, at 100 µM, abolishes it and prevents the proarrhythmic effect of tyramine, noradrenaline and isoproterenol. Inhibition of Na^+^ current is an important mechanism of antiarrhythmic drug action (Srivatsa, Wadhani & Singh, 2002) and higher concentration of resveratrol, such as the 100 µM used in this work, reduces Na^+^ current by 50% (Chen, Su & Hung, 2007; Chen et al., 2009). Given that a concentration 40 µM of the typical Na^+^ current inhibitor lidocaine, which inhibits this current by 50% (Scholz et al., 1998), abolished the proarrhythmic effect of IBMX and mimicked the effect of resveratrol in our study, the most probable mechanism responsible for the antiarrhytmic effect of resveratrol would be the inhibition of Na^+^ current. The concentration 100 µM of resveratrol, although reported to produce other beneficial effects such as the inhibition of proliferation and apoptosis of a variety of tumor cells (Lin, 2005), is above the levels observed in humans given therapeutic doses of resveratrol. However, such a concentration could be reached by overdose, suggesting that, in this case, resveratrol would not enhance cardiac automaticity but may even protect against the ventricular arrhythmias induced by other inotropic agents given concomitantly.

The sinoatrial node rate is also controlled by cAMP levels, which, in turn, are regulated by high PDE activity, which activates protein kinase A-dependent local subsarcolemmal ryanodine receptor Ca^+2^ release and an inward Na^+^/Ca^+2^ exchange (for review see Vinogradova & Lakatta, 2009). The inhibition of PDE activity reduces cAMP hydrolysis and enhances cAMP levels and the sinoatrial node rate (Vinogradova & Lakatta, 2009; Merino, Quesada & Hernández-Cascales, 2015), just as IBMX, an inhibitor of PDE activity, does in the present work. In contrast, resveratrol has no effect on the sinoatrial node rate, possibly due to its inhibitory effect on subsarcolemmal ryanodine receptor Ca^+2^ release (Liu et al., 2005), which may counteract its inhibitory effect on PDE activity.

Resveratrol is a widely distributed stilbenoid found in several plants, including berries, peanuts and, in particularly high concentrations, grape skins and red wine (Varela-López et al., 2015). The cardioprotective ability of resveratrol has been extensively studied since it first attracted attention following the finding that the consumption of red wine, which contains relatively high levels of resveratrol, reduces the incidence of mortality and morbidity from coronary heart disease (Raj, Zieroth & Netticadan, 2015). The broad spectrum of cardioprotective benefits conferred by resveratrol includes a reduction in ischemia reperfusion injury, the inhibition of low density lipoprotein oxidation and platelet aggregation, antioxidant, anti-inflammatory and antiapoptosis effects, antiarrhythmic effects, as well as the ability to suppress sympathetic neural remodelling after myocardial infarction (Xin et al., 2010). Heart failure, is a widely spread debilitating disease with a poor prognosis but in which resveratrol seems to have beneficial effects (Zordoky, Robertson & Dyck, 2015; Sung & Dyck, 2015). Impaired contractile function and cardiac arrhythmias are common findings in these patients (Yancy et al., 2013). Inotropic agents (beta adrenoceptor agonists or phosphodiesterase inhibitors) are still recommended in a significant proportion of patients with severe systolic dysfunction, low blood pressure and significantly depressed cardiac output (Yancy et al., 2013). These drugs may improve cardiac contractility but also induce ventricular arrhythmias and sudden death in some patients (Overgaard & Dzavík, 2008). The results of this work indicate that the administration of resveratrol in combination with beta agonist agents may well increase their inotropic effects but not their proarrhythmic effects. Also, in contrast to other PDE inhibitors, resveratrol does not produce sinoatrial tachycardia. These are interesting findings of potential clinical relevance, although further research is necessary to confirm this possibility.

##  Supplemental Information

10.7717/peerj.3113/supp-1 Supplemental Information 1 Noradrenaline–Resveratrol interaction on ventricular inotropism Raw data exported from the contractile effect of noradrenaline applied for data in Fig. 1, Fig. 7 and Table 1.

10.7717/peerj.3113/supp-2 Supplemental Information 2 Tyramine–Resveratrol interaction on ventricular inotropism Raw data exported from the contractile effect of tyramine applied for data in Fig. 2 and Table 1.

10.7717/peerj.3113/supp-3 Supplemental Information 3 Isoproterenol–Resveratrol interaction on ventricular inotropism Raw data exported from the contractile effect of isoproterenol applied for data in Fig. 1 and Table 1.

10.7717/peerj.3113/supp-4 Supplemental Information 4 Interaction of IBMX with either noradrenaline, tyramine or isoproterenol on ventricular inotropism Raw data exported from the contractile effect of noradrenaline, tyramine and isoproterenol in combination with IBMX applied for data in Fig. 5 and Table 1. The inotropic effect of IBMX alone showed in Fig. 4 is also indicated.

10.7717/peerj.3113/supp-5 Supplemental Information 5 Effects of noradrenaline, tyramine or isoproterenol on ventricular automaticity Raw data showing changes in ventricular rate (beats min^−1^) induced by noradrenaline, tyramine and isoproterenol alone or combined with resveratrol or IBMX, applied for data in Fig. 6.

10.7717/peerj.3113/supp-6 Supplemental Information 6 Effects of resveratrol and IBMX on sinoatrial rate Raw data showing the effects of resveratrol and IBMX on sinus node applied to Fig. 8.

## Additional Information and Declarations

### Data Availability

The following information was supplied regarding data availability: The raw data has been supplied as a Supplementary File.
