# Supplementary material for: Resveratrol enhances the inotropic effect but inhibits the proarrhythmic effect of sympathomimetic agents in rat myocardium
Source: PeerJ. 2017 Mar 30;5:e3113. doi: 10.7717/peerj.3113 (PMC5376116; doi:10.7717/peerj.3113)
Supplement: Supplemental Information 1 — Raw data exported from the contractile effect of noradrenaline applied for data in Fig. 1, Fig. 7 and Table 1. [file peerj-05-3113-s001.doc]

**NORADRENALINE**

| μM | 1 | 2 | 3 | 4 | 5 |
| --- | --- | --- | --- | --- | --- |
| 0.01  0.03  0.1  0.3  1  3  10 | 3  8  6  59  75  85  85 | 0  2  25  35  75  100  100 | 2  5  33  66  86  100  100 | 0  3  31  55  63  83  83 | 0  5  17  45  70  100  100 |

**NORADRENALINE + RESVE (10** μM)

| μM | 1 | 2 | 3 | 4 | 5 |
| --- | --- | --- | --- | --- | --- |
| 0.01  0.03  0.1  0.3  1  3  10 | 7  20  60  86  100  100  100 | 5  10  43  78  85  85  85 | 8  10  50  83  83  83  83 | 3  20  55  88  88  88  88 | 5  12  28  72  100  100  100 |

**NORADRENALINE+VEHICLE**

| μM | 1 | 2 | 3 |
| --- | --- | --- | --- |
| 0.01  0.03  0.1  0.3  1  3  10 | 5  12  37  63  87  95  95 | 0  2  22  45  73  100  100 | 0  0  10  40  60  85  85 |

**NORADRENALINE + RESVE (100** μM)

| nM | 1 | 2 | 3 | 4 |
| --- | --- | --- | --- | --- |
| 3  10  30  100  300 | 5  25  75  100  100 | 0  11  90  100  100 | 0  33  66  100  100 | 0  28  71  100  100 |

**NORADRENALINE+LIDOCAINE**

| μM | 1 | 2 | 3 |
| --- | --- | --- | --- |
| 0.03  0.1  0.3  1  3  10 | 5.8  29.4  64.7  76  76  76 | 3.9  17  49  75  95  95 | 4.5  25  53  67  90  90 |
