# Supplementary material for: Resveratrol enhances the inotropic effect but inhibits the proarrhythmic effect of sympathomimetic agents in rat myocardium
Source: PeerJ. 2017 Mar 30;5:e3113. doi: 10.7717/peerj.3113 (PMC5376116; doi:10.7717/peerj.3113)
Supplement: Supplemental Information 2 — Raw data exported from the contractile effect of tyramine applied for data in Fig. 2 and Table 1. [file peerj-05-3113-s002.doc]

**TYRAMINE**

| μM | 1 | 2 | 3 | 4 | 5 | 6 |
| --- | --- | --- | --- | --- | --- | --- |
| 1  3  10  30  100  300  1000 | 0  6  17  35  59  55  55 | 0  6  19  37  62  60  60 | 0  0  14  28  57  56  56 | 0  0  6  19  25  30  30 | 0  0  7  14  36  43  43 | 0  0  9  27  45  54  54 |

**TYRAMINE + RESVE (10** μM)

| μM | 1 | 2 | 3 | 4 |
| --- | --- | --- | --- | --- |
| 1  3  10  30  100  300  1000 | 0  25  62  75  75  73  73 | 0  10  30  40  50  60  50 | 0  7  33  47  53  53  53 | 0  13  41  53  59  62  62 |

**TYRAMINE + VEHICLE**

| μM | 1 | 2 | 3 |
| --- | --- | --- | --- |
| 1  3  10  30  100  300  1000 | 0  7  10  25  39  40  43 | 0  4  12  30.5  42  46  50 | 0  0  15  28.5  47  57  56 |

**TYRAMINE + RESVE (100** μM)

| μM | 1 | 2 | 3 | 4 | 5 |
| --- | --- | --- | --- | --- | --- |
| 0.1  0.3  1  3  10  30 | 5  18  75  100  100  100 | 12  25  75  100  100  100 | 0  15  60  80  100  100 | 14  29  57  72  86  86 | 0  8  20  40  60  60 |
