# Supplementary material for: Resveratrol enhances the inotropic effect but inhibits the proarrhythmic effect of sympathomimetic agents in rat myocardium
Source: PeerJ. 2017 Mar 30;5:e3113. doi: 10.7717/peerj.3113 (PMC5376116; doi:10.7717/peerj.3113)
Supplement: Supplemental Information 3 — Raw data exported from the contractile effect of isoproterenol applied for data in Fig. 1 and Table 1. [file peerj-05-3113-s003.doc]

**ISOPROTERENOL**

| nM | 1 | 2 | 3 | 4 |
| --- | --- | --- | --- | --- |
| 1  3  10  30  100  300  1000 | 0  0  12  37  62  81  87 | 0  0  12  25  50  87  100 | 0  0  0  9  54  82  100 | 0  10  40  60  80  100  100 |

**ISOPROTERENOL + RESVE (10** μM)

| nM | 1 | 2 | 3 | 4 |
| --- | --- | --- | --- | --- |
| 1  3  10  30  100  300  1000 | 0  13  25  33  75  100  100 | 0  7  33  73  85  100  100 | 6  19  38  63  82  88  88 | 0  10  40  70  80  80  80 |

**ISOPROTERENOL+VEHICLE**

| nM | 1 | 2 | 3 |
| --- | --- | --- | --- |
| 1  3  10  30  100  300  1000 | 0  0  3  17  50  83  87 | 0  5  17  40  65  95  95 | 0  0  20  40  68  100  100 |

**ISOPROTERENOL + RESVE (100** μM)

| nM | 1 | 2 | 3 | 4 |
| --- | --- | --- | --- | --- |
| 1  3  10  30  100  300 | 42  72  100  100  100  100 | 0  0  38  75  100  100 | 37  56  82  100  100  100 | 15  43  71  100  100  100 |
