# Supplementary material for: Resveratrol enhances the inotropic effect but inhibits the proarrhythmic effect of sympathomimetic agents in rat myocardium
Source: PeerJ. 2017 Mar 30;5:e3113. doi: 10.7717/peerj.3113 (PMC5376116; doi:10.7717/peerj.3113)
Supplement: Supplemental Information 4 — Raw data exported from the contractile effect of noradrenaline, tyramine and isoproterenol in combination with IBMX applied for data in Fig. 5 and Table 1. The inotropic effect of IBMX alone showed in Fig. 4 is also indicated. [file peerj-05-3113-s004.doc]

**NORADRENALINE+IBMX**

| nM | 1 | 2 | 3 | 4 |
| --- | --- | --- | --- | --- |
| 1  3  10  30  100  300  1000 | 0  3  25  45  80  85  85 | 0  0  33  80  100  100  100 | 0  0  20  60  85  95  95 | 0  10  40  70  93  100  100 |

**TYRAMINE + IBMX**

| μM | 1 | 2 | 3 | 4 |
| --- | --- | --- | --- | --- |
| 1  3  10  30  100  300 | 11  28  56  72  77  77 | 8  37  70  80  85  85 | 15  45  68  75  82  82 | 12  30  55  70  80  80 |

**ISOPROTERENOL + IBMX**

| nM | 1 | 2 | 3 | 4 |
| --- | --- | --- | --- | --- |
| 1  3  10  30  100  300 | 3  9  45  81  100  100 | 20  43  70  85  85  85 | 25  50  66  83  83  83 | 7  42  78  92  85  85 |

**INCREASE BASAL CONTRACTILITY**

**IBMX (30** μM)

|  | 1 | 2 | 3 | 4 |
| --- | --- | --- | --- | --- |
| % | 18 | 27 | 20 | 22 |
