# Supplementary material for: Resveratrol enhances the inotropic effect but inhibits the proarrhythmic effect of sympathomimetic agents in rat myocardium
Source: PeerJ. 2017 Mar 30;5:e3113. doi: 10.7717/peerj.3113 (PMC5376116; doi:10.7717/peerj.3113)
Supplement: Supplemental Information 5 — Raw data showing changes in ventricular rate (beats min−1) induced by noradrenaline, tyramine and isoproterenol alone or combined with resveratrol or IBMX, applied for data in Fig. 6. [file peerj-05-3113-s005.doc]

**VENTRICULAR RATE**

**NORADRENALINE+ RESVE**

|  | 1 | 2 | 3 | 4 |
| --- | --- | --- | --- | --- |
| Control  NA  (0.1 μM)  ∆ V.R  RESVE  (100μM)  NA  (3 μM) | 15  87  72  0  0 | 1  16  15  0  0 | 7  38  31  0  0 | 53  79  26  0  0 |

**NORADRENALINE**

**+ IBMX**

|  | 1 | 2 | 3 | 4 |
| --- | --- | --- | --- | --- |
| Control  NA  (0.1 μM )  ∆ V.R  IBMX  **(**30μM)  ∆ V.R | 27  43  31  180  153 | 35  56  21  93  58 | 3  40  40  145  142 | 8  27  19  80  72 |

**NORADRENALINE + VEHICLE**

|  | 1 | 2 | 3 |
| --- | --- | --- | --- |
| Control  NA  (0.1 μM )  ∆ V.R  VEHICLE | 3  27  24  23 | 54  93  39  38 | 25  57  32  35 |

**TYRAMINE + RESVE**

|  | 1 | 2 | 3 | 4 | 5 |
| --- | --- | --- | --- | --- | --- |
| Control  TYR  (30 μM)  ∆  RESVE  (100μM)  TYR  (100 μM) | 55  93  37  0  0 | 14  25  13  0  0 | 0  26  26  0  0 | 0  30  30  0  0 | 46  60  14  0  0 |

**TYRAMINE + IBMX**

|  | 1 | 2 | 3 | 4 |
| --- | --- | --- | --- | --- |
| Control  TYR  (30 μM)  ∆ V.R  IBMX  (30 μM)  ∆ V.R | 2  23  21  180  178 | 24  85  51  210  186 | 0  37  37  170  133 | 3  50  47  200  147 |

**TYRAMINE + VEHICLE**

|  | 1 | 2 | 3 |
| --- | --- | --- | --- |
| Control  TYR  (30 μM)  ∆  VEHICLE | 30  63  33  35 | 0  25  25  20 | 3  34  31  27 |

**ISOPROTERENOL + RESVE**

|  | 1 | 2 | 3 | 4 | 5 |
| --- | --- | --- | --- | --- | --- |
| Control  ISO  (0.1 μM)  ∆ V.R  RESVE  (100μM)  ISO  (1 μM) | 1  63  62  0  0 | 20  70  50  0  0 | 0  35  35  0  0 | 36  85  49  0  0 | 7  63  56  0  0 |

**ISOPROTERENOL + IBMX**

|  | 1 | 2 | 3 | 4 |
| --- | --- | --- | --- | --- |
| Control  ISO  (0.1 μM)  ∆ V.R  IBMX  (**30**μM)  ∆ V.R | 12  43  31  180  168 | 0  56  56  87  87 | 37  125  88  170  133 | 3  50  47  107  104 |

**ISOPROTERENOL + VEHICLE**

|  | 1 | 2 | 3 |
| --- | --- | --- | --- |
| Control  ISO  (0.1 μM)  ∆ V.R  VEHICLE | 0  47  47  53 | 53  84  31  28 | 5  73  68  65 |

∆ **V.R: Increase ventricular rate**
