# Supplementary material for: Resveratrol enhances the inotropic effect but inhibits the proarrhythmic effect of sympathomimetic agents in rat myocardium
Source: PeerJ. 2017 Mar 30;5:e3113. doi: 10.7717/peerj.3113 (PMC5376116; doi:10.7717/peerj.3113)
Supplement: Supplemental Information 6 — Raw data showing the effects of resveratrol and IBMX on sinus node applied to Fig. 8. [file peerj-05-3113-s006.doc]

**ATRIAL RATE**

**RESVERATROL**

|  | 1 | 2 | 3 | 4 | 5 | 6 | 7 |
| --- | --- | --- | --- | --- | --- | --- | --- |
| Control  10 μM  100 μM | 314  325  298 | 363  365  345 | 338  343  329 | 325  329  325 | 347  347  354 | 237  221  220 | 249  244  253 |

**IBMX**

|  | 1 | 2 | 3 | 4 | 5 | 6 |
| --- | --- | --- | --- | --- | --- | --- |
| Control  1 μM  10 μM  100 μM | 315  328  345  415 | 294  315  358  396 | 241  262  303  370 | 250  290  380  430 | 264  284  321  386 | 261  281  302  350 |

**RESVERATROL VEHICLE (DMSO)**

|  | 1 | 2 | 3 |
| --- | --- | --- | --- |
| Control  10 μM  100 μM | 308  312  300 | 297  290  280 | 327  325  337 |
